# Stabilizing working memory in spiking networks with biologically plausible synaptic dynamics

**DOI:** 10.1186/1471-2202-15-S1-P157

**Published:** 2014-07-21

**Authors:** Alexander Seeholzer, Moritz Deger, Wulfram Gerstner

**Affiliations:** 1 School of Life Sciences, Brain Mind Institute and School of Computer and Communication Sciences, École polytechnique fédérale de Lausanne, 1015 Lausanne EPFL, Switzerland

## 

Behavior often requires remembering continuously structured information, e.g. positions in the visual field, over delay periods of up to seconds. How can a neural circuit reliably store this information using biophysical mechanisms that work on timescales of milliseconds? Recurrently connected networks with continuous attractors [1,2] provide a solution by creating a self-sustained bump-shaped neural activity profile that can be positioned along a continuous degree of freedom. This freedom of position, however, renders the activity bump highly sensitive to the sources of variability expected in cortical networks: low connection probabilities, suboptimal synaptic weights or heterogeneity of neuronal parameters. These can lead to a quick drift of the bump position and thus detrimental loss of acuity of the encoded memory. Short-term facilitation (STF) stabilizes drift in continuous attractors, as shown recently in simplified neural network models [3,4].

In neurons STF acts by dynamically regulating neurotransmitter release, mainly onto NMDA channels, however these simplified models neglect detailed synaptic integration mechanisms. It is thus unclear whether comparable stabilization can be achieved with biologically plausible synaptic dynamics, which limit the effects of STF, like activity dependent saturation of NMDA receptors [2] and conductance based synaptic transmission.

**Figure 1:**
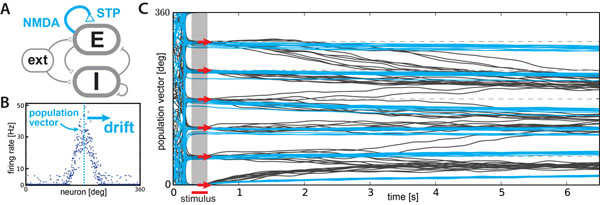
**A** Network of excitatory (E) and inhibitory (I) spiking neurons (schematic). Recurrent E connections show short-term plasticity (STP) and complex NMDA dynamics. **B** Bump-shaped activity profile of the excitatory population, encoding an angle close to 180° in the population vector. Motion of this encoded angle is called drift. **C** Population vectors storing 6 different angles (red arrows) in working memory (10 repetitions each) at a connectivity of 50%. Without STP the acuity of stored angles is quickly lost due to angular drift (gray lines), whereas STF can stabilize these traces (blue lines).

To address this issue we combined two influential classes of models: spiking models of cortical networks with conductance based synapses and detailed dynamics of NMDA receptors [2], and models of short-term dynamics of presynaptic transmitter release [5] (see Figure 1). We derive analytical predictions for the amount of drift expected from the different sources of variability described above and investigate the extent to which STF can alleviate their detrimental influence, allowing us to place constraints on the combinations of network and synapse properties. Thus, we demonstrate the extent to which STF alone can stabilize the temporal evolution of continuous attractors in biologically plausible networks and clarify whether additional stabilization mechanisms are necessary to make these models candidates for functional working memory.
